# Dengue epidemic in China before 1978

**DOI:** 10.1186/s40249-024-01243-y

**Published:** 2024-09-26

**Authors:** Xiang Guo, Haiyang Chen, Ruifeng Lin, Xiaohua Liu, Meng Li, Liu Ge, Wenting Deng, Rangke Wu, Xiaohong Zhou

**Affiliations:** 1 Institute of Tropical Medicine, Department of Pathogen Biology, School of Public Health, Southern Medical University, Guangdong Provincial Key Laboratory of Tropical Disease Research, Key Laboratory of Prevention and Control for Emerging Infectious Diseases of Guangdong Higher Institutes, Key Laboratory of Infectious Diseases Research in South China, Ministry of Education, Guangzhou, 510515 Guangdong China; 2 Department of Rheumatology and Immunology, Nanfang Hospital, Southern Medical University, Guangzhou, China; 3 Department of Traditional Chinese Internal Medicine, School of Traditional Chinese Medicine, Southern Medical University, Guangzhou, China; 4 The School of Foreign Studies, Southern Medical University, Guangzhou, 510515 China

**Keywords:** Dengue virus, Comprehensive review, Epidemics, Ancient literature

## Abstract

Dengue, an acute febrile disease transmitted by *Aedes* mosquitoes, is caused by the dengue virus (DENV), presenting a formidable challenge to global public health. By examining clues from ancient Chinese books and conducting a comprehensive review, this study elucidates the characteristics of potential dengue epidemics in China prior to 1978. This evidence indicates that China may not have experience dengue epidemics before 1840. During 1840–1949, however, it experienced a noticeable dengue occurrence and prevalence in the 1870s, 1920s, and 1940s. Then from 1949 to 1978, only sporadic reports were accounted. The disparity in the frequency of dengue occurrences across three time periods suggests that the persistent characteristic of dengue epidemics in China primarily arises from imported cases resulting from international exchanges, subsequently leading to local outbreaks influenced by global epidemic trend. This research offers a novel perspective on retrospectively examining the historical trajectory of dengue epidemics and provides valuable insights into exploration of DENV epidemic patterns.

**Graphical Abstract:**

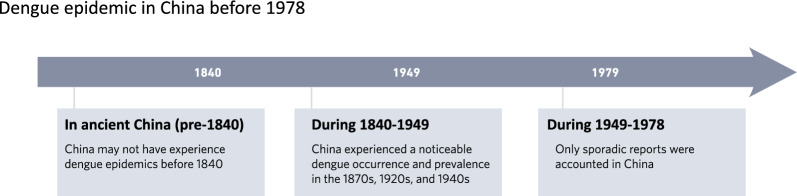

**Supplementary Information:**

The online version contains supplementary material available at 10.1186/s40249-024-01243-y.

## Background

Dengue, an important acute febrile disease, is transmitted by *Aedes aegypti* or *Ae. albopictus* mosquitoes and is caused by infection with dengue virus (DENV) [1, 2]. Historically, DENV spread across tropical areas in the eighteenth and nineteenth centuries [2]. However, in the following twentieth and twenty-first centuries, this disease gained powerful momentum for epidemic activity due to radical growth and massive migration of human population, extensive urbanization, modern transportation systems, global trade, climate change, and the lack of effective mosquito control [2, 3]. Specifically, the comprehensive revival of regional economies and urban development following World War II, coupled with the expansion of civil aviation in the 1970s, accelerated global communication and broadened the regions susceptible to dengue outbreaks [2, 3]. These factors have contributed to the drastic increase of dengue cases globally. Currently, an estimated 390 million new cases occur annually worldwide, affecting a population of approximately 2.5 to 4 billion and posing a formidable threat to global public health [4]. Consequently, the rapid spread of the disease has also resulted in a series of invasive outbreaks in temperate countries, such as Italy, Spain, and northern China [5–8].

In May 1978, a dengue outbreak, caused by DENV-4, was reported in Foshan City, Guangdong Province, China [9]. Subsequent years witnessed recurrent outbreaks with a substantial number of DENV infection cases, particularly in 1978, 1980, and 1986 in Hainan and Guangdong Province [9]. From 1990 to 2012, a discernible pattern of milder epidemics and periodic outbreaks emerged, especially in 1995, 2002, and 2006, when the number of infections exceeded one thousand in number, with notably high infection rates documented [10–12]. The extensive outbreak during the period of 2013–2014 marked a shift, with multiple serotypes of DENVs co-circulating in China, predominantly in Guangdong and Yunnan provinces [13, 14]. Despite the extensive epidemic reports in China, the majority of historical accounts of prevalent diseases in China only extend back to the 1978 dengue outbreak in Foshan, Guangdong [10, 11, 15]. While historical records document numerous epidemics with symptoms similar to dengue fever analyses on the pre-1978 documents are scarce.

This study aims to shed light on the history of dengue epidemics in China prior to 1978, by examining evidence from ancient Chinese literature and conducting comprehensive reviews. The findings identify potential epidemiological events that could elucidate the pre-1978 history of dengue in China.

## Dengue epidemic in ancient China (pre-1840)

In ancient China, there was no recognition of contemporary fever-transmitting pathogens such as dengue and Zika: hence there was no specific name for dengue. However, there are some potential clues of early dengue records in the descriptions of symptoms or epidemic features, including ‘yellow disease’ for skin manifestations, ‘red exanthem’ or ‘speckled exanthem’ for exanthem, and ‘water poison’ for vectors documented in traditional Chinese works.

A commonly cited statement in current published literature is: The earliest known clinical and epidemiological account of a possible dengue-like illness can be found in a Chinese encyclopedia documenting disease symptoms and treatments, which was first published during the Jin Dynasty (AD 317–420) (Additional file 1: Table S1) [3, 16, 17]. However, upon tracing the volume seven of ‘Handbook of Prescriptions for Emergencies’, it was found that the original description is far from the clinical symptoms of dengue. Meanwhile, descriptions of ‘water poison’ can be found in ‘General Treatise on Causes and Manifestations of All Diseases’ (Additional file 1: Table S1), which closely resemble schistosome cercarial dermatitis. In this works, ‘water poison’ (schistosome cercariae) enters through the skin, damaging it and resulting in the appearance of wheals and exanthem accompanied by itching [18, 19].

In ‘General Treatise on the Causes and Manifestations of All Diseases’ and ‘Taiping Holy Prescriptions for Universal Relief’, there are another symptom description of ‘yellow disease’ that closely resemble the clinical manifestations of dengue, such as ‘sore and painful eyes, nasal bone pain, stiffness in both arms and neck, and acute pain in the waist and back’ (Additional file 1: Table S1). However, the descriptions state that the initial symptoms are characterized by a ‘hollow yellow complexion’ and ‘patients with yellow disease often experience constipation and quick urination, but the disease does not lead to patient’s death’. These symptoms more closely resemble the medical condition known as jaundice (Additional file 1: Table S1).

Similarly, the relevant literature does not mention any clinical features resembling dengue that could help to differentiate it from other diseases like scarlet fever and childhood exanthem (Additional file 1: Table S1). Ancient works reveal few descriptions of symptoms, similar to those of dengue, and even though there are some, the descriptions appear often ambiguous and could potentially relate to other diseases for differential diagnosis. Given the extensive scope and documentation practices of Chinese ancient literature, if there had been significant dengue cases, records similar to those documented in 1873 Penghu County Record (Additional file 1: Table S1) would probably have been uncovered. From this perspective, we can infer that dengue likely did not break out in ancient China.

## Dengue epidemic in China during 1840–1949

China has undergone drastic social changes and wars in this period, accompanied by frequent foreign trade and warfare, which have led to the increasingly frequent occurrence of dengue as recorded in both Traditional Chinese Medicine records and Modern Medicine records (Table 1, Additional file 1: Table S1). During 1840–1949, China experienced a noticeable trend of concentrated reports on dengue occurrence and prevalence in the 1870s, 1920s, and 1940s (Table 1, Additional file 1: Table S1). In the 1870s, dengue cases were predominantly observed in urban areas with active maritime trade along the southeastern coast, such as Xiamen in 1873 and Macau in 1874 [20]). Subsequently, during the 1920s, there was an expansion of reported outbreaks. Amidst World War II in the 1940s, large-scale dengue epidemics occurred among urban communities in China, including coastal city Hangzhou and even inland city Hankou (Table 1). Taiwan experienced an unprecedentedly severe dengue epidemic in 1942, with the documented cases reaching several staggering 114,722 individuals. Scholars posit that the magnitude of DENV infections in Taiwan in that year might have affected approximately five million people (Table 1).

**Table 1:** Dengue epidemic in China before 1978 in modern medicine literature

Year	Location	References
1870s	Taiwan	[21]
1873	Xiamen	[22]
1874	Macau	[20]
1889	Taiwan	[23]
1902–1903	Taiwan	[23–26]
1915	Taiwan	[23]
1922	Penghu, Taiwan	[27]
1924	Taiwan	[23, 28, 29]
1927	Kaohsiung	[23]
1928–1929	Guangzhou, Xiamen, Hangzhou, Ningbo, Shanghai, Taiwan, Hong Kong	[22]
1931	Taiwan	[23]
1942	Taiwan	[23]
1942–1945	Shanghai, Zhejiang, Jiangsu, Fujian, Guangdong, Taiwan, Wuhan	[22]
1975	Yunnan	[30]
1976	Beijing	[31]
1970s	Guangxi	[32]

## Dengue epidemic in China during 1949–1978

During the period from 1949 to 1978, China experienced a prolonged period of low dengue prevalence, lasting nearly four decades, only a few cases reported in Yunnan Province in 1975 and Beijing City in 1976 (Table 1).

## Discussion

From our analysis of the historical stages of dengue prevalence in China, it becomes clear that foreign trade policies have been an important factor in shaping the spread of dengue. Prior to 1840 and from 1949 to 1978, China’s engagement in foreign mobility and trade activities was limited. In contrast, between 1840 and 1949, dengue cases were predominantly reported in early commercial port cities, with few exceptions. Despite a global trend towards increased prevalence in the 1950s, China did not experience a corresponding surge. It is evident that foreign mobility and trade activities have played a pivotal role in the spread of dengue in China. This discernible pattern persisted until 1978, when China began to implement progressively more open policies. This period marked by an escalation in dengue prevalence across geographical areas within China, reflecting the country’s openness to foreign trade and mobility. The notable surge in dengue cases reported in China during the period from 1840 to 1949 mirrors dengue pandemics during the same periods. For instance, reports of dengue/dengue-like fever in trade port cities coincided with the so-called dengue global pandemic of 1870–1873 [16, 20].

In our previous study, we proposed a three-stages model to characterize the invasion, colonization, and diffusion patterns of DENV [33]. Over the past two decades, evidence strongly suggests that dengue epidemics in Guangzhou have evolved from a simple importation pattern to a complex epidemic pattern [33].

This speculation can also be further explored from several perspectives: First, historical climate conditions indicate that the temperature in China between 1120 and 1900 was approximately 1 °C lower than current temperatures, which likely hindered local transmission and outbreaks of dengue, as inferred from Zhu Kezhen’s curve. Second, the presence of vector mosquitoes in ancient China remains uncertain, as genetic studies have not conclusively provided evidence for their existence. However, it is impractical to definitely conclude that there were no cases of dengue in ancient China due to the challenges in thoroughly examining all ancient books and Traditional Chinese Medicine gazetteers.

## Conclusions

This paper systematically presents the key characteristics of dengue epidemic in China prior to 1978. It argues that there is limited evidence suggesting dengue outbreaks in ancient China before 1840. During 1840–1949, concentrated reports of dengue occurrence and epidemic are noted in the 1870s, 1920s, and 1940s, while only sporadic reports existed during the period of from 1949 to 1978. The disparity in the frequency of dengue occurrences across these time periods suggests that the persistent characteristic of dengue epidemics in China primarily arises from imported cases resulting from international exchanges, subsequently leading to local outbreaks influenced by global epidemic trend. This study offers a novel perspective on retrospectively examining the history of dengue epidemics and provides valuable references for exploring patterns of dengue outbreaks.

## Supplementary Information

Additional file 1. **Table S1. **Dengue clinical symptom descriptions found in Traditional Chinese Medicine classics, local chronicles, and ancient textual records

Additional file 2. **Text S1**. Literature search strategy in this study.

## Data Availability

Not applicable.
