# Supplementary material for: Dengue epidemic in China before 1978
Source: Infect Dis Poverty. 2024 Sep 26;13:69. doi: 10.1186/s40249-024-01243-y (PMC11425873; doi:10.1186/s40249-024-01243-y)
Supplement: Supplementary file 1 — Additional file 1. Table S1. Dengue clinical symptom descriptions found in Traditional Chinese Medicine classics, local chronicles, and ancient textual records [file 40249_2024_1243_MOESM1_ESM.docx]

**Table S1.** Dengue clinical symptom descriptions found in Traditional Chinese Medicine classics, local chronicles, and ancient textual records

| **Clue word*** | **Year** | **Sources*** | **Translation in English** | **Translation in modern Chinese** | **Original description in Traditional Chinese Medicine literature** |
| --- | --- | --- | --- | --- | --- |
| Water poison  [水毒] | 317-420 | Handbook of Prescriptions for Emergencies  [肘后备急方] | Water poison, also known as *Zhongxi*, *Zhongsa*, or water disease, is a condition with symptoms similar to those caused by archer-poisonous insects but without any insects being found. The initial symptoms of this disease include fever and chills, slight headaches, pain around the ocular orbit, dysphoria, limbs tremors, severe pain in the joints and bones, and muscle spasms. There may also be fever and drowsiness which increases at night but decreases during the day, and deadly coldness in hands and feet. After the second or third day of the illness worms appear in abdomen and attack downward towards the perineum area, and causing sores around the anus that are neither painful nor itchy. Therefore, the patient with the disease does not feel any abnormalities until emergence of these symptoms which delays timely treatment. After six or seven days of illness onset, the perineum become festering, and the insects invaded the *Five Zang organs*, resulting in predominant heat and causing dysphoria and diarrhea and fecal incontinence. After eight or nine days, experienced doctors believe that it is difficult to treat it, suggesting that attention should be paid to treating perineum as soon as possible at the onset of illness. If the sore looks like freshly cut meat, the most dangerous *Yang* toxin occurs; if the sore looks like worn-out fish teeth, *Yin* toxin becomes fatal even although it is not so urgent. | 水中毒，又称中溪、中洒、水病，其症状类似由射工虫引起的疾病，但实际上在体内未能发现射工虫。该病初始症状为恶寒发热，轻微头痛，眼眶疼痛，烦闷懊恼，手脚发冷，四肢震颤，关节和骨骼剧烈疼痛强硬。肌肉痉挛，嗜睡，症状夜间加重白天减轻。在患病的第三天之后，出现好似有虫从腹部向下攻击，并在肛门周围引起不痛不痒也不觉得发冷的疮。因为患有这种疾病的患者直到发现会阴部生疮时才会感到异常，所以治疗不及时。发病六七天后，会阴部溃烂，好似有虫侵犯五脏，体内热盛，烦躁不安，大便下痢不禁。经过八九天之后，经验丰富的医生尚且无法医治，建议在发病初期就应该重视会阴部的症状。如果疮的颜色如鲜肉，病情危急，称为阳毒；如果看似磨损的鱼齿，虽病情稍缓，基本上也会是致命的，称为阴毒。 | 水毒中人，一名中溪，一名中洒，一名水病。似射工而无物，其诊法。初得之恶寒，头微痛，目注疼，心中烦懊，四肢振淅，骨节皆强。筋急，但欲睡，旦醒，暮剧。手逆冷，三日则复生虫食下疮，不痛不痒不冷。人觉视之乃知，不即疗。过六七日下部脓溃，虫食五脏，热极烦毒。注下不禁，八九日，良医不能疗，觉得急，当深视下部。若有疮，正赤如截肉者为阳毒，最急。若疮如蠡鱼齿者为阴毒，犹小缓，要皆煞人。 |
|  | 610 | General Treatise on the Causes and Manifestations of All Diseases  [诸病源候论] | Water poisoning is prevalent in the eastern and southern regions of the Three Wu areas (now referred to Jiangsu, Zhejiang, Anhui, Guangdong provinces in China), caused by infection with toxic insects found in mountain valleys and streams. The initial symptoms of this disease include aversion to cold, slight headaches, dysphoria, stiffness and pain in the back, joints and bones, and knee pain. Otherwise, fever and drowsiness worsen at night but improved during the day. Additionally, there is a sensation of deadly coldness in hands and feet extending up to the elbows and knees. These symptoms may also cause non-painful or itchy festering sores in the perineum area, combined with diarrhea and fecal incontinence accompanied by blood that is colored like a decaying liver, strong desire to eat, and raving. | 水毒发病流行于三吴东南地区，即如今江苏、浙江、安徽和广东省一带。病因为感染生长在山谷和溪流中的有毒昆虫。其初始症状可见恶寒，轻微头疼，眼眶疼痛，烦躁懊恼，腰背部及关节骨骼强硬，双膝疼痛；或出现低热伴有嗜睡，症状白天减轻夜间加重，手脚发冷且逆行至肘膝关节；或出现会阴部长疮，患者不觉痛痒，会阴部脓肿溃烂，大便下痢，排泄的血颜色如腐坏的肝，纳差，胡言乱语。 | 流行于三吴以东及南（现江、浙、皖、粤一带）；因中山谷溪源处恶虫毒所致；初病可见恶寒，头微痛，目眶疼，心内烦懊，腰背骨节皆强，两膝疼，或翕翕热但欲睡，旦醒暮剧，手足指逆冷至肘膝，或可有下部生疮，不痛不痒，脓溃，湿热下注，不食狂语，下血物如烂肝等。 |
| Yellow disease  [黄病] | 610 | General Treatise on the Causes and Manifestations of All Diseases  [诸病源候论] | Yellow disease is characterized by symptoms such as fever, pantalgia, and a sallow complexion. After seven or eight days, the illness progresses with experiencing high fever, diarrhea and fecal incontinence accompanied by blood that is colored like a decaying liver, and lower abdominal distention and spasms. Individuals with these symptom may also experience dryness and pain in eyes, soreness in nasal bone, shoulders, arms, and neck, as well as back spasms. Constipation is a common symptom among people with this disease. If they can urinate normally, their condition is not severe; however, if they cannot, their stool will become firmer and more compact leading to epigastric oppression. | 黄病，该病症状为全身疼痛、发热、面色蜡黄，发病七八天后高热，下痢如肝血色，小腹胀痛痉挛。有的人眼睛干涩疼痛，鼻骨痛，肩颈腰背疼痛，这也是黄病的症状。黄病患者大多排便困难，如果能使其小便排泄正常则不担心病情危急。不可使便秘情况加重，否则心烦胀闷。 | 黄病者，一身尽疼发热，面色洞黄，七八日后壮热，口里有血，当下之，如猪肝状，其人小腹满急。若其人眼睛涩疼，鼻骨痛，两膊及项强，腰背急，即是患黄也。黄病多大便涩，但令得小便快，即不虑死。不令大便多涩，涩即心胀不安。 |
|  | 960-1127 | Taiping Holy Prescriptions for Universal Relief  [太平圣惠方] |  |  |  |
| Red exanthem [红痧] | 1774 | Shen’s Zunsheng Book  [沈氏尊生书] | Red exanthem, faint red rashes resembling skin rashes, appear on the skin's surface above the muscles. Despite the mild symptoms, it is advised to avoid consuming hot alcohol and water. External scraping is recommended. | 红痧，该病症状为在肌肉上方的皮肤表面出现隐约可见的红点。虽然症状较轻，但不可以接触热酒热水。可用刮痧外治。 | 曰红痧，皮肤隐隐红点，如相似，痧在肌表，感受虽浅，热酒热汤，亦不可犯，外用刮。 |
|  | 1882 | Shibing Lun  [时病论] | People from the southern region may have insufficient *Qi*. Accidental contact with fecal matter or filth can cause immediate abdominal discomfort and pain. This condition is referred to as red exanthem, which is a misspelling of the word sand. Some individuals may also develop a faintly visible red rash on the cutaneous surface overlying the musculature, thus referred to as red exanthem. | 生活在南方地区的人体内正气不充足，偶然接触粪土秽浊之气，会立即出现腹痛闷胀，这种疾病被称为痧。痧是沙的讹字。有些人可能在肌肉组织上的皮肤表面出现隐约可见的红色皮疹，因此被称为红痧。 | 南方之人，体**气**不实，偶触粪土沙秽之气，即腹痛闷乱，名之曰**痧**，即沙字之讹也。又有肤隐红点，一如 疹，此痧在肌表，为**红痧**也。 |
| Peaceful illness  [太平病] | 1873 | Penghu County Record (In Taiwan province)  [澎湖县志] | This winter, the people suffered from an unusual disease. The disease began with a mild soreness in the waist and limbs, rapidly progressing to complete paralysis and an inability to ambulate with severe bone and muscle pain. Some people contracted the disease on the road, unable to walk independently but had to be carried home. Those who consumed warm herbal decoctions died, while those who took cold-decoctions recovered. Therefore, the fatality rate were relatively low. After recovery, the patients would remain weak in their limbs for one to two months, requiring a long time to regain some strength. This unusual disease is commonly known as the "peaceful illness", affecting both men and women. | 今年冬天，百姓罹患一种不寻常的疾病。这种疾病开始时患者觉得腰部和四肢轻度疼痛，而后迅速发展为全身瘫软无法行走，伴有严重的骨骼和肌肉疼痛。有的人在路上感染了这种病，只能搀扶回家没办法独立行走。服用性热的汤剂病情会加重，服用性味寒凉的汤剂能治愈，病死的不多。治愈后一两个月内患者觉得四肢无力，症状很久才能稍微减轻，这种情况俗称平安病，患这种病的男女症状相同。 | 是冬，民得異疾，其始自覺腰肢微酸，旋即遍身癱軟，不能行動，筋骨疼痛異常，有途次得疾，未及抵家而扶腋以歸者。服熱劑者死，惟服冷可愈（癒），故死者尚少。愈（癒）後一、二月尚覺手足無力，久始暫瘥，俗謂之「平安病」。廳屬男婦皆然，亦異症也。 |
| Summer plague  [暑瘟] | 1937 | Juegang County Record (In Jiangsu province)  [倔港县志] | In the 26th year of the Republic of China, a disease called "dengue fever" in Western Medicine was epidemic. There was a prevalence of infection among all the members of some families, and even doctors fell ill one after another. The summer plague lasted for more than 50 days, leading to a mortality rate of infants as high as 100%. | 民国二十六年，西医名“登革症”流行，有的全家染病，连医生都相继病倒，暑瘟流行长达50多天，婴儿死亡率高达100%。 | |
| Shanghai fever  [上海病] | 1940 | Shengongjian  (Pers.comm) | The disease is originally called Dengue in Western Medicine and commonly transliterated as ‘Dengue fever’ in Chinese. It presents with initial symptoms including fever, bone soreness and pain, discomfort in the chest and hypochondrium, nausea, and a rapid pulse. After two or three days, the patients experience red rash spots appear on the limbs. As the rash spots erupt, body temperature gradually decreases, and other symptoms also subside. | 在今年秋天，西方命名为Dengue而音译成登革热的一种疾病在本地区流行严重。该病初起症状为发热、骨骼疼痛、胸胁不适、恶心，脉数增加。发病后两三天，四肢出现鲜红色的皮疹红点，疹点透发之后，体温逐渐降低至正常，各种症状也减轻消失。 | 西籍原名Dengue, 译音通称登革热, 今秋本埠甚为流行，初起发热，骨楚疼痛，胸胁作恶，脉数增加，病后二、三日，四肢发现鲜红色的疹点，疹点透发后，则体温渐降，各症亦减退。 |

*The Chinese text enclosed within square brackets represents the original title or name of Traditional Chinese Medicine literature.The italicized terms signify the standardized terminology specific to Traditional Chinese Medicine, as referenced from the authoritative publication, "WHO International Standard Terminologies on Traditional Chinese Medicine."
