# Supplementary material for: Dengue epidemic in China before 1978
Source: Infect Dis Poverty. 2024 Sep 26;13:69. doi: 10.1186/s40249-024-01243-y (PMC11425873; doi:10.1186/s40249-024-01243-y)
Supplement: Supplementary file 2 — Additional file 2. Text S1. Literature search strategy in this study. [file 40249_2024_1243_MOESM2_ESM.docx]

**Text S1**. Literature search strategy in this study.

For examining clues from ancient Chinese books, we conducted a literature search in Chinese databases including ancient literature like Duxiu - ‘knowledge part’ (https://www.duxiu.com/) and Dingxiu (http://www.ding-xiu.com/), as well as Chinese modern journal databases China National Knowledge Infrastructure Databases (CNKI, http://www.cnki.net) and Wanfang (http://g.wanfangdata.com.cn/). The search included terms like clues of early dengue records ‘水毒’ or ‘黄病’ or ‘上海病’ or ‘太平病’ or ‘瘴气’ or ‘暑瘟’ or ‘红砂’ or ‘斑痧’ or ‘痧气’ or ‘骨痛’ or ‘热症’. The literature review followed specific selection criteria, involving independent searches by authors, resolution of conflicts through expert consultation, and a meticulous screening process based on title, abstract, and full-text to exclude irrelevant studies. Additionally, other relevant records identified through references of selected reports or expert recommendations were included to ensure that all eligible records were included for descriptive analyses.
